# Inducible, Dose-Adjustable and Time-Restricted Reconstitution of *Stat1* Deficiency *In Vivo*

**DOI:** 10.1371/journal.pone.0086608

**Published:** 2014-01-29

**Authors:** Nicole R. Leitner, Caroline Lassnig, Rita Rom, Susanne Heider, Zsuzsanna Bago-Horvath, Robert Eferl, Simone Müller, Thomas Kolbe, Lukas Kenner, Thomas Rülicke, Birgit Strobl, Mathias Müller

**Affiliations:** 1 Institute of Animal Breeding and Genetics, University of Veterinary Medicine Vienna, Vienna, Austria; 2 University Center Biomodels Austria (BIAT), University of Veterinary Medicine Vienna, Vienna, Austria; 3 Clinical Institute of Pathology, Medical University of Vienna, Vienna, Austria; 4 Ludwig Boltzmann Institute for Cancer Research, Vienna, Austria; 5 Institute of Pharmacology and Toxicology, University of Veterinary Medicine Vienna, Vienna, Austria; 6 Department of Internal Medicine I, Institute for Cancer Research, Comprehensive Cancer Center, Medical University of Vienna, Vienna, Austria; 7 Department IFA-Tulln, Biotechnology in Animal Production, University of Natural Resources and Applied Life Sciences, Tulln, Austria; 8 Institute of Laboratory Animal Science, University of Veterinary Medicine Vienna, Vienna, Austria; Karolinska Institutet, Sweden

## Abstract

Signal transducer and activator of transcription (STAT) 1 is a key player in interferon (IFN) signaling, essential in mediating host defense against viruses and other pathogens. STAT1 levels are tightly regulated and loss- or gain-of-function mutations in mice and men lead to severe diseases. We have generated a doxycycline (dox) -inducible, FLAG-tagged *Stat1* expression system in mice lacking endogenous STAT1 (i.e. *Stat1^ind^* mice). We show that STAT1 expression depends on the time and dose of dox treatment in primary cells and a variety of organs isolated from *Stat1^ind^* mice. In bone marrow-derived macrophages, a fraction of the amount of STAT1 present in WT cells is sufficient for full expression of IFN-induced genes. Dox-induced STAT1 established protection against virus infections in primary cells and mice. The availability of the *Stat1^ind^* mouse model will enable an examination of the consequences of variable amounts of STAT1. The model will also permit the study of STAT1 dose-dependent and reversible functions as well as of STAT1's contributions to the development, progression and resolution of disease.

## Introduction

Signal transducer and activator of transcription (STAT) 1 is involved in various cellular mechanisms such as proliferation, differentiation and apoptosis. It is part of the so-called Janus kinase (JAK)/STAT pathway, which is activated by the binding of interferons (IFNs) and other cytokines and growth factors to their cognate receptors. STAT1 is an essential component of the signaling pathways for all three types of IFN. IFN type I (mainly IFNα's and β) and IFN type III (IFNλs) activate the IFN stimulated gene factor 3 (ISGF3), which binds IFN stimulated response elements (ISRE). IFN type II (IFNγ) activates STAT1 homodimers, which bind to IFNγ-activated sites (GAS). Recruitment of STAT1 complexes to ISRE or GAS results in the induction of specific but overlapping sets of IFN induced genes (ISGs) [1]–[3].

In unstimulated cells, STAT1 generally resides in the cytoplasm in the form of inactive dimers. Activation of JAKs enables them to phosphorylate their receptors and thereby allow the recruitment of STAT proteins, which are subsequently phosphorylated on key tyrosine residues. Phosphorylation of STAT1 dimers on tyrosine 701 leads to a conformational change, translocation into the nucleus and initiation of transcription [4], [5]. Additional phosphorylation on serine 727 takes place in the nucleus and is essential for full transcriptional activity [6]–[8].

Evidence is accumulating that the availability of STAT1 determines the responsiveness to IFN. Cellular STAT1 levels are maintained and enhanced through an autoregulatory loop that is dependent on IFN type I [9]. Accordingly, mice gene-targeted in components of the IFN type I production and response cascade (e.g. *IFN*β, IFNα receptor 1 [*Ifnar1*], tyrosine kinase 2 [*Tyk2*], *Stat2* and *Irf3*) show reduced levels of STAT1 [10]–[13]. Knowledge of the basal amounts of STAT1 required for cellular functionality is sparse, although an increase of STAT1 in *Ifnar1*-deficient fibroblasts restores IFNγ-induced expression of target genes and antiviral activity [9]. It has also been shown that IFN type I-increased STAT1 levels counteract the STAT4-driven IFNγ production of activated NK cells [14]. The ratio of STAT1:STAT4 is also an important determinant of the CD8^+^ T cell response upon virus infection [15]. Analysis of *Stat1*-deficient T cells has revealed that STAT1 strongly effects the activation of Th1 lineage-specific enhancers, thus contributing to chromatin-shaping processes [16].

The importance of STAT1 is underlined by the effects of germline loss- and gain-of-function mutations in mice and men. Null mutations of *Stat1* result in high susceptibility to bacterial and viral pathogens [17]–[21]. In humans, partial loss of STAT1 activity leads to mycobacterial and viral diseases, although in contrast to cases completely lacking STAT1 such patients are curable [17], [18], [22]–[24]. *Stat1*-deficient mice kept under specific pathogen-free conditions do not succumb to infection and can be studied. STAT1 has been shown to have tumor-suppressive functions against spontaneous and induced solid cancers, including breast cancer [25]–[30]. In contrast, STAT1 has a tumor-promoting function in leukemogenesis [31] and progressed melanoma [32]. *STAT1* gain-of-function mutations were discovered in human patients diagnosed with chronic mucocutaneous candidiasis caused by perturbed cytokine responses and resulting in impaired function of Th17 cells [33]–[38]. To date, gain-of-function studies of STAT1 in experimental systems have been restricted to the effects of the *in vitro* expression of constitutively active STAT1 (STAT1C) [39], [40].

Genome-wide association studies reveal that common disease-associated polymorphisms are enriched in STAT1 promoter binding sites and that deregulated levels and/or activation of STAT1 are closely associated with the development of auto-immune/-inflammatory disorders and cancers [41]. Consistently with this finding and the results of loss-of-function studies in mice [42], STAT1 expression is often lost in human breast cancer biopsies and STAT1 has been discussed as a predictive marker for breast cancer therapy [25], [43]. Remarkably, STAT1 gain-of-function mutations and the concomitant increase in activity levels have been shown to distinguish disease phenotypes and outcomes in myeloproliferative neoplasms [44]. Balanced STAT1 activity is thus indispensable for functional immunity and prevention of tumor development in mice and men [18].

To date, there is no *in vivo* model available to study the impact of dose- and time-dependent STAT1 functions in a reversible manner. Tetracycline- or derivative-controlled gene expression systems have proven to be the ideal tool for reversibly inducible gene expression in mammals. We report here the generation of a doxycycline (dox) – inducible *Stat1* (*Stat1^ind^*) mouse model based on the Tet-On system [45]. We show that expression of inducible STAT1 depends on the time and dose of dox treatment in primary cells and tissues/organs. Detailed biochemical analysis proves that transgenic STAT1 can drive ISG expression and mediate antiviral activity *in vitro* and *in vivo*. Furthermore, we show that spatially and temporally balanced STAT1 expression is essential for host defense against virus infection.

## Materials and Methods

### Ethics Statement

Mice were housed under specific pathogen-free conditions according to FELASA guidelines. All animal experiments were discussed and approved by the Ethics and Animal Welfare Committee of the University of Veterinary Medicine Vienna, conform to the guidelines of the national authority (the Austrian Federal Ministry for Science and Research) as laid down in §8ff of the Animal Science and Experiments Act (Tierversuchsgesetz – TVG; refs BMWF-68.205/0204-C/GT/2007; BMWF-68.205/0210-II/10b/2009, BMWF-68.205/0243-II/3b/2011) and according to the guidelines of FELASA and ARRIVE. To assess the distress of the animals during infection experiments, a scoring system was established. Based on this, health status and behavior of the animals were controlled by trained staff (participants of FELASA B training course) every 3–4 hours during 7 a.m. and 7 p.m. In order not to disturb the circadian rhythm of the animals there was no monitoring after 7 p.m. Human endpoint was conducted by cervical dislocation if death of the animals was to be expected during the following hours.

### Cloning, Gene Targeting and Mice

For the establishment of *Stat1^ind^* mice we used a 2^nd^ generation Tet-On system based on a site-specific recombination system in embryonic stem (ES) cells [46], [47]. The KH2 (C57BL/6×129Sv) ES cells express the M2 reverse tetracycline (tet)-controlled transactivator (M2rtTA) under the control of the endogenous *Rosa26* promoter and a neomycin resistance cassette flanked by flippase recognition target (*frt*) sites inserted downstream of the collagen type I alpha 1 (*Col1a1*) locus. We targeted a construct carrying the tet operator (tetO) followed by a *FLAG*-tagged *Stat1a* cDNA (Genbank accession number NM_009283) into the *Col1a1* locus of KH2 ES cells by *frt*/flippase-mediated recombination. *Stat1a* cDNA was PCR-amplified from C57BL/6N bone marrow-derived macrophages (BMMΦs) mRNA with the primers 5′ AGC GTC TCG AAT TCC CAC CAT GTC ACA GTG GTT CGA GCT TCA G 3′ and 5′ ACG AAT TCG AGA CGT TAA TTA ATT AAG CTA CTT GTC ATC GTC GTC CTT GTA ATC TAC TGT GCT CAT CAT ACT GTC 3′. Thereby, Esp3I restriction sites resulting in EcoRI overhangs were introduced to both ends of the amplified *Stat1a* cDNA. Additionally, the primer overhangs included a *FLAG* tag at the 3′ end of *Stat1* (amino acid sequence: DYKDDDDK). The modified *Stat1a* cDNA was cloned via EcoRI (Thermo Scientific) into the vector *pBS31′-RGBpA* directly under the control of a doxycycline-inducible promoter element [46] and designated *pBS31-Stat1a-FLAG*. The vector and a vector encoding the flippase (*pCAGGS-FLP* [46]) were co-transfected into KH2 ES cells using Nucleofector Technology (Amaxa Inc.). ES cells were treated with 140 μg/ml hygromycin B (ROTH) to select clones that have undergone site-specific recombination, which converts the cells from G418- to hygromycin B-resistant. Three independent hygromycin B-resistant ES cell clones showed correct and single integration of the construct, as verified by Southern blot analysis (Fig. S1). ES cells were injected into C57BL/6N blastocysts and resulting chimeras were crossed to *Stat1*-deficient mice (B6N.129*Stat1^tm1Dlv^*, referred to here as *Stat1^−/−^* [19]). We obtained triple-mutant mice heterozygous for (i) the *M2rtTA* in the *Rosa26* locus, (ii) the dox-inducible *Stat1* construct in the *Col1a1* locus and (iii) the targeted mutation of the endogenous *Stat1* locus. Heterozygous mice were intercrossed to obtain homozygous triple-mutant B6N;129P2-*Stat1^tm1Dlv^*, Gt(ROSA)26Sor^tm1(rtTA*M2)Jae^, *Col1a1^tm1(tetO-Stat1)Biat^* mice (referred to here as *Stat1^ind^* mice). Wild-type (WT) mice and homozygous triple-mutant *Stat1^ind^* mice were bred separately in a B6N;129Sv mixed genetic background. In parallel, mice were backcrossed to C57BL/6N and BALB/c by speed congenics [48]. All experiments were performed using WT and *Stat1^ind^* mice in a B6N;129Sv mixed genetic background and *Stat1^−/−^* mice in C57BL/6N. Mice were fed with doxycycline (dox; Sigma) at concentrations of 0.2 or 1 mg/ml supplemented with 10 mg/ml sucrose *via* drinking water.

### Cell Culture and Reagents

ES cells, primary embryonic fibroblasts (PEFs) and BMMΦs were cultivated as previously described [49]. IFNβ and IFNγ were purchased from Calbiochem and Millipore. For *in vitro* experiments, cells were treated with dox dissolved in water at concentrations ranging from 25 ng/ml to 10 μg/ml.

### PCR, RT-qPCR and Southern Blot

Genotyping of *Stat1^ind^* mice and cells was performed using three different PCR protocols for each modified locus. *Col1a1*: *Col1*-fwd: 5′ TGC TCG CAC GTA CTT CAT TC 3′; *Col1*-rev: 5′ CAA CCT GGT CCT CCA TGT CT 3′; *Hygro*-fwd: 5′ ACT GTC GGG CGT ACA CAA AT 3′ (amplicon size for WT: 255 bp; *Stat1^ind^*: 500 bp). *Rosa26*: *Rosa26*-fwd: 5′ AAA GTC GCT CTG AGT TGT TAT 3′; *Rosa26*-rev: 5′ GGA GCG GGA GAA ATG GAT ATG 3′; *Rosa26*-mut: 5′ GCG AAG AGT TTG TCC TCA ACC 3 (amplicon size for WT: 625 bp; *Stat1^ind^*: 350 bp). *Stat1*: *Stat1*-fwd: 5′ TGT TCT TCC TCT CCC TTT AGG A 3′; *Stat1*-rev: 5′ CAC ATG GAC TGC TGT GCT TT 3′; *Stat1*-mut: 5′ GGG TGG GGT GGG ATT AGA TA 3 (amplicon size for WT: 335 bp; *Stat1^ind^*: 372 bp). The cycling conditions were 95°C for 5 min and 35 cycles of 95°C for 30 sec, 60°C for 30 sec and 72°C for 30 sec.

RT-qPCR was performed as described [50]. Quantitative mRNA concentrations were analyzed with linear models (ANOVA and t-tests) using genotype and treatment as factors. The following primers and probes were used. *Stat1*-fwd: 5′ GAT CAG CTG CAA ACG TGG TTC 3′; *Stat1*-rev: 5′ GCT TTT TAA GCT GCT GAC GGA 3′; *Stat1*-fam: 5′CCA TTG TTG CAG AGA CCC TGC AGC A 3′; *Mx1*-fwd: 5′ CCT GGA GGA GCA GAG TGA CAC 3′; *Mx1*-rev: 5′ GGT TAA TCG GAG AAT TTG GCA A 3′; *Mx1*-fam: 5′ TTA AGG CTG GAT GAG GCT CGG CAG A 3′; *Irf1*-fwd: 5′ CCG AAG ACC TTA TGA AGC TCT TTG 3′; *Irf1*-rev: 5′ GCA AGT ATC CCT TGC CAT CG 3′; *Irf1*-fam: 5′ CAG TCT GAG TGG CAG CCG ACA CAC A 3′.

Southern blot analysis was performed using a 5′-external *Col1a1* probe of 400 bp isolated with PstI/XhoI (Thermo Scientific) digestion [46]. Genomic DNA of gene-targeted clones was digested with SpeI (Thermo Scientific).

### Western Blot and Electrophoretic Mobility Shift Assay (EMSA)

Protein lysates and Western blots were performed as described [50]. Additional antibodies were purchased from Bethyl Laboratories (ECS directed against the FLAG-tag) and Santa Cruz (β-tubulin). EMSAs were performed as described [49].

### Immunohistochemistry

Immunohistochemistry was performed on formalin-fixed and paraffin-embedded (FFPE) heart, liver and spleen tissue samples. Antigen retrieval was achieved by incubation in citric buffer pH 6 for 20 min at 100°C. For the detection of STAT1, rabbit polyclonal anti-STAT1 antibody (from Santa Cruz in Fig. 4; from Cell Signaling in Fig. S6) was applied. Antibody binding was visualized using peroxidase-conjugated secondary antibody.

### Flow Cytometry

Freshly isolated splenocytes from WT and *Stat1^ind^* mice were stained for 20 min at 4°C with anti MHC class I-specific antibody H-2Db-PE in combination with anti-T cell-specific antibody CD4-FITC (both eBioscience). For analysis, at least 5×10^4^ events were collected on a FACSCanto II^TM^. Data were analyzed using FACSDiva^TM^ software (both BD Biosciences). Mean fluorescence intensities were analyzed with linear models (ANOVA and t-tests) using genotype and treatment as factors and resulting p-values reported.

### Viruses

VSV (Indiana strain) was in antiviral assays in PEFs as described [20]. Crystal violet stained cells were solubilized in 100 μl of a 50/50 mixture of 0.1 M NaH_2_PO_4_ and 50% EtOH. Crystal violet intensity was measured at 595 nm using an Epoch Microplate Spectrophotometer (Biotec). Mean number of survivng cells from three independent experiments were calculated against each unstimulated genotype. Statistics was performed with a linear model (ANOVA) using genotype as factor.

Survival experiments with mice using VSV and EMCV were performed as described [51]. Survival data were analyzed using Kaplan-Meier estimators.

## Results

### Time- and dose-dependent expression of doxycycline-induced STAT1 in *ex vivo* cultured cells from *Stat1^ind^* mice

**Figure 1:**
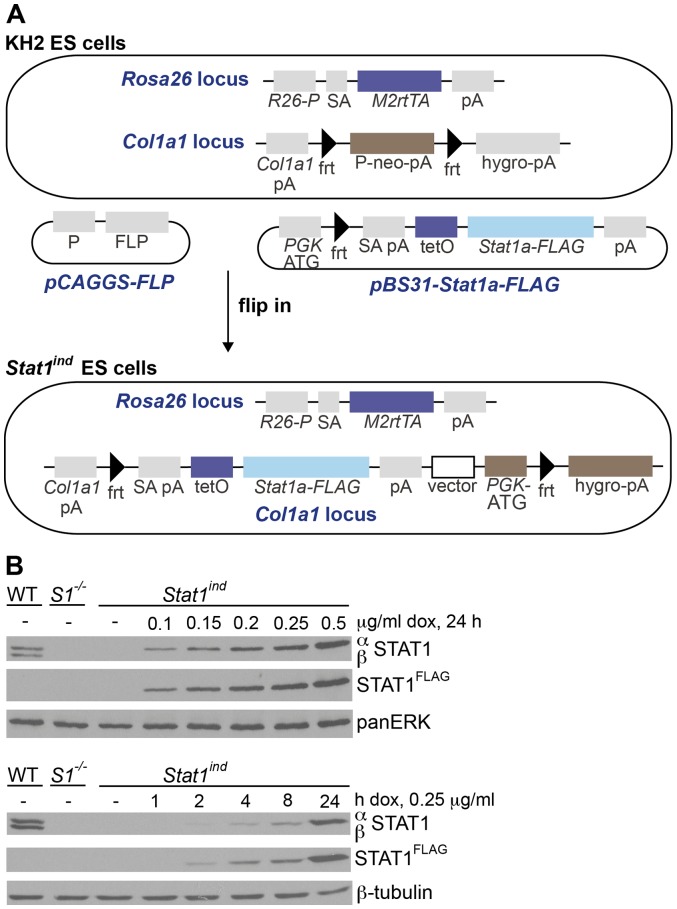
Schematic representation of *Stat1^ind^* ES cells and induced STAT1^FLAG^ production in primary cells. **A**) The upper panel shows the organization of KH2 ES cells harboring the M2rtTA transactivator under the control of the endogenous *Rosa26* promoter followed by a polyadenylation signal (polyA). The *Col1a1* locus contains a neomycin-resistance cassette flanked by *frt* sites, followed by a promoter-less hygromycin resistance cassette. The vector *pCAGGS-FLP* encodes the flippase. The vector *pBS31-Stat1a-FLAG* consists of the tetracycline operator (tetO) followed by a *FLAG*-tagged *Stat1a* cDNA and a polyA sequence. Additionally, it codes for a *PGK* promoter followed by an *frt* site. After co-transfection of the two vectors and correct flp-in into the *Col1a1* locus, the *Stat1^ind^* ES cells are depicted in the lower panel. *R26-P*: *Rosa26* promoter; SA: splice acceptor; M2rtTA: M2 reverse tetracycline-controlled transactivator; pA: polyadenylation signal; *frt*: flippase recognition target; P: promoter; neo: neomycin resistance cassette; hygro: hygromycin resistance cassette; FLP: flippase; tetO: tetracycline operator. **B**) BMMΦs were isolated from WT, *Stat1^−/−^* (*S1^−/−^*) and *Stat1^ind^* (homozygous for *M2rtTA* and *Stat1a-FLAG* alleles backcrossed to *Stat1^−/−^*) mice and treated with different amounts of dox for 24 h (upper panel) or for different times with 0.25 μg/ml dox (lower panel). Western blots were performed to analyze expression of STAT1; membranes were reprobed with an antibody against the FLAG tag. Note that *Stat1^ind^* cells only express STAT1α, whereas both STAT1 isoforms are detected in WT cells. As loading control, panERK or β-tubulin were used. Results are shown from one representative of at least three independent experiments.

STAT1 exists in two isoforms, full length STAT1α and STAT1β, which is truncated at the carboxy terminal and thus lacks part of the transactivation domain [52]. We used *Stat1a*-cDNA to generate inducible *Stat1* mice. KH2 embryonic stem cells expressing the M2rtTA under the control of the endogenous *Rosa26* promoter were targeted with a dox-inducible, *FLAG*-tagged *Stat1a-*cDNA downstream of the *Col1a1* locus by *frt*/flippase-mediated site-specific recombination (Fig. 1A). Three independent clones showed correct and single integration of the construct, as proven by Southern blot analysis (Fig. S1). Transgenic STAT1 (termed STAT1^FLAG^) could be detected in all three ES cell clones after administration of dox for 24 h using a FLAG-tag specific antibody. Importantly, we did not observe any leakiness of the system, i.e. there was no STAT1^FLAG^ expression in the absence of dox (Fig. S2A).

The gene-targeted ES cells were suitable for the generation of inducible *Stat1*-transgenic mice. ES cells were injected into C57BL/6N blastocysts and the resulting chimeras were bred with *Stat1*-deficient C57BL/6N mice. Heterozygous offspring were intercrossed to obtain homozygous triple-targeted mice for (i) the tet-activator in the *Rosa26* locus, (ii) the dox-responsive *Stat1*-construct and (iii) the targeted mutation in the endogenous *Stat1* locus. Homozygous triple-targeted mice were designated *Stat1^ind^* and used for all experiments in a B6N;129Sv mixed genetic background with matching WT controls. Additionally, mice were backcrossed to C57BL/6N and BALB/c by speed congenics [48].

In the absence of dox, no STAT1^FLAG^ could be detected in bone marrow-derived macrophages (BMMΦs) isolated from *Stat1^ind^* mice. STAT1^FLAG^ expression was dependent on the dose of dox applied (Fig. 1B, upper panel). STAT1^FLAG^ induction started after 2 h of dox treatment and reached WT levels after 24 h using 0.25 μg/ml dox (Fig. 1B, lower panel). STAT1^FLAG^ was also expressed in primary embryonic fibroblasts (PEFs; Fig. S2B) and splenocytes (Fig. S2C) in a time- and dose-dependent manner. Only in PEFs was low basal STAT1^FLAG^ detectable, indicating some degree of tissue-specific leakiness of the *Col1a1* and/or *Rosa26* loci. In contrast to previous attempts to generate inducible *Stat1*-transgenic mice [49], the Tet-On system gave rise to viable inducible *Stat1* transgenics. Importantly, STAT1 levels similar to, above or below those of WT STAT1 can readily be achieved *ex vivo*, so the system is suitable for studying the dose effects of preformed STAT1 in various cell types.

### STAT1^FLAG^ is phosphorylated and binds to DNA upon IFN treatment *in vitro*

**Figure 2:**
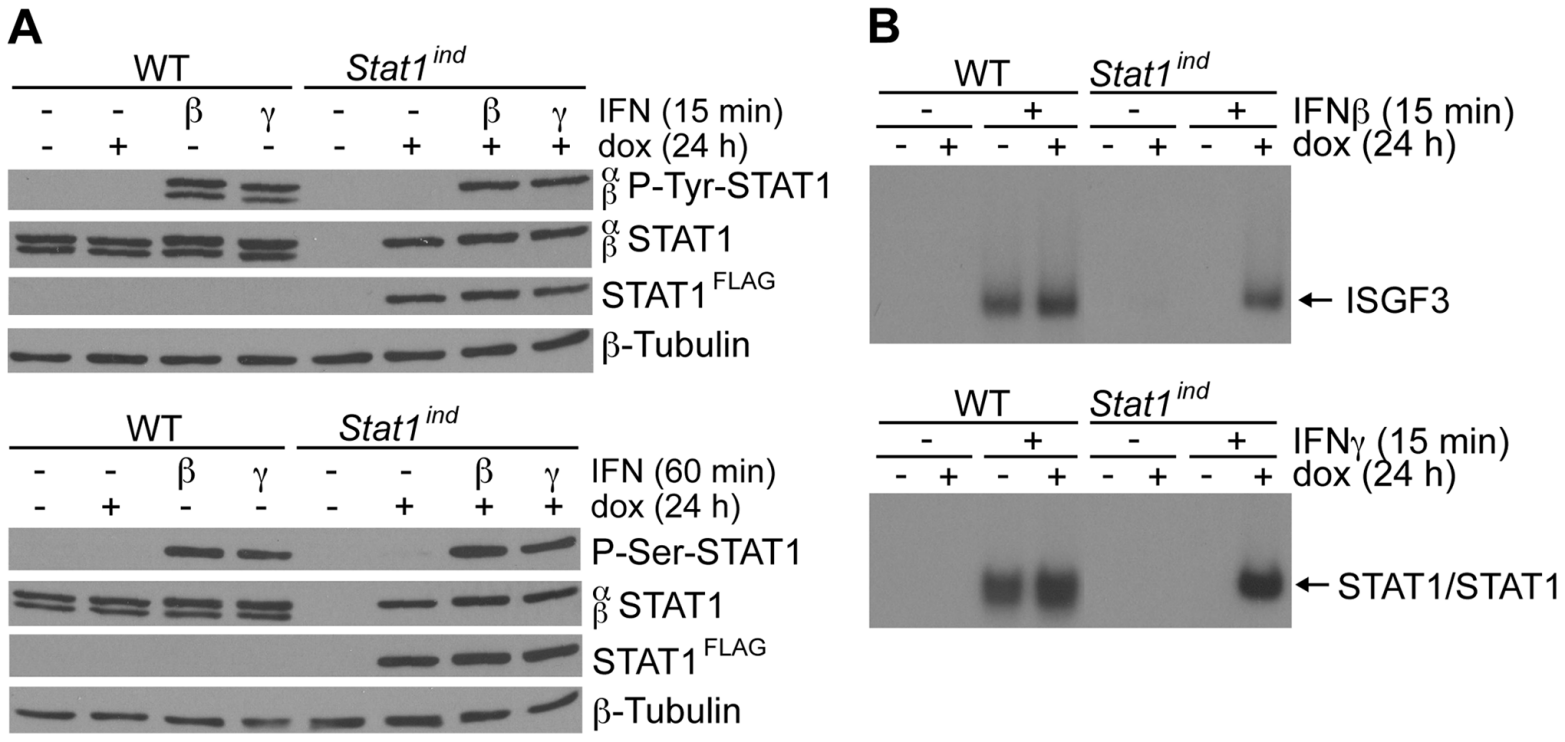
Phosphorylation and DNA binding of IFN-activated STAT1^FLAG^ in BMMΦs. BMMΦs were isolated from WT and *Stat1^ind^* mice. Cells were pretreated with 0.25 μg/ml dox for 24 h and stimulated with 100 U/ml IFNβ or IFNγ for 15 or 60 min or left untreated. Results are shown from one representative out of three independent experiments. **A**) Protein lysates were subjected to Western blot analysis for tyrosine 701-phosphorylated STAT1 (P-Tyr-STAT1; upper panel) or serine 727-phosphorylated STAT1 (P-Ser-STAT1; lower panel). Membranes were reprobed with antibodies against STAT1 and FLAG. Loading was controlled by reprobing the membranes with β-tubulin. **B**) EMSAs were performed to analyze binding of ISGF3 to ISRE (upper panel) or STAT1/STAT1 to GAS elements (lower panel).

In dox-treated BMMΦs isolated from *Stat1^ind^* mice, STAT1^FLAG^ was phosphorylated on tyrosine 701 and serine 727 at levels similar to WT STAT1 after 15 or 60 min treatment with IFNβ or IFNγ (Fig. 2A). Electrophoretic mobility shift assays showed that in response to stimulation with IFNβ, the STAT1^FLAG^ protein forms ISGF3 and binds to ISRE sites; in response to stimulation with IFNγ it forms STAT1 homodimers and binds to GAS sites. Again, DNA binding activity was comparable to that in WT cells (Fig. 2B). The data demonstrate the biochemical functionality of STAT1^FLAG^. In accordance with previous reports, the addition of short sequences to the C-terminus of STAT1 does not affect the protein's ability to dimerize, to be phosphorylated and to bind DNA [5], [53].

### STAT1^FLAG^ is a fully functional transcription factor and mediates antiviral activity

**Figure 3:**
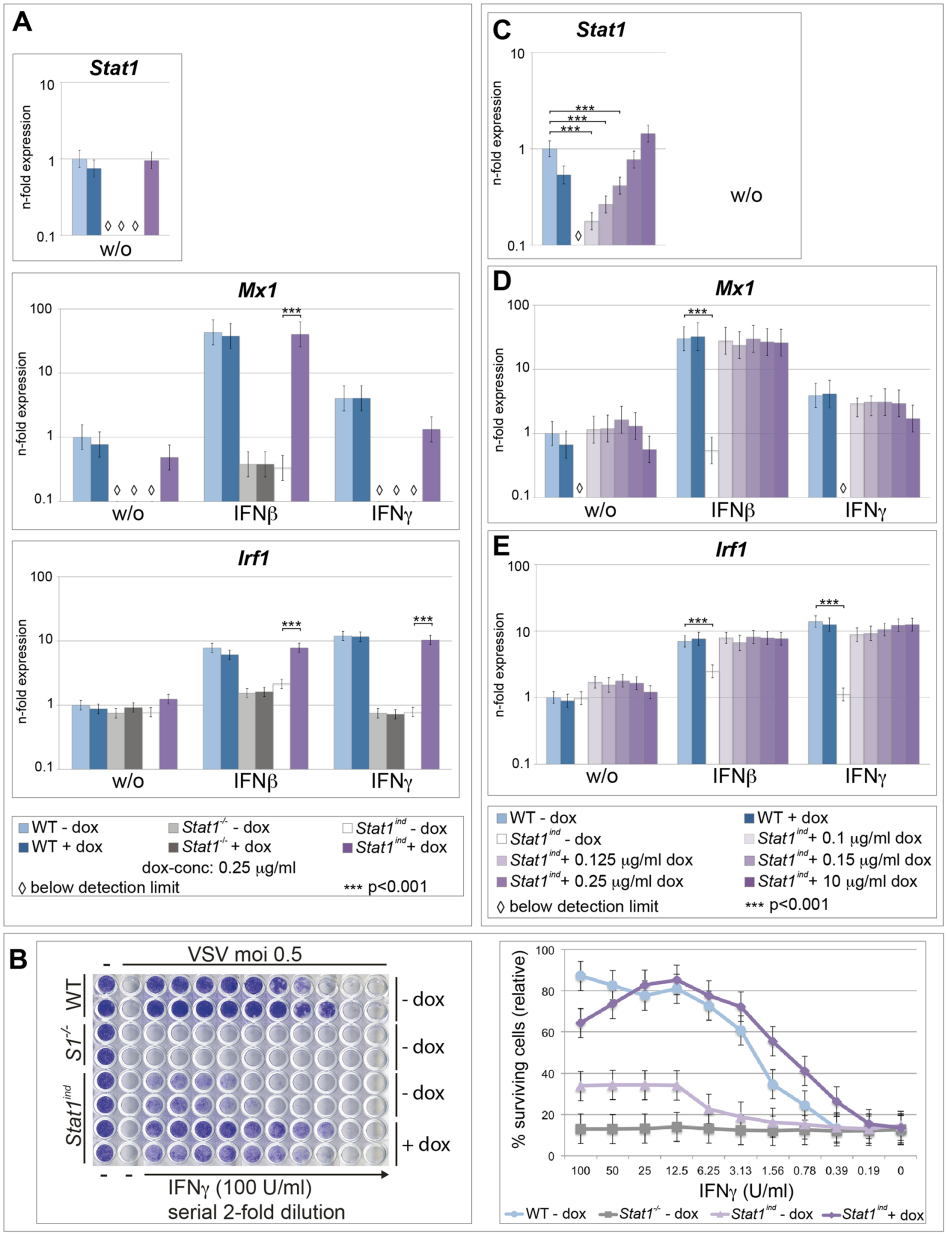
IFN-induced transcription of *Mx1* and *Irf1* at low or WT cellular amounts of STAT1^FLAG^ and antiviral activity of STAT1^FLAG^. **A**) BMMΦs of various genotypes were pretreated with 0.25 µg/ml dox for 24 h and stimulated with 100 U/ml IFNβ or IFNγ for 4 h or left untreated (w/o). RNA was isolated and cDNA used to analyze expression of *Stat1* (upper panel), *Mx1* (middle panel) and *Irf1* (lower panel) mRNA. *Ube2d2* was used for normalization and expression values were calculated relative to untreated WT cells. Results are shown as mean values ± SE from three independent experiments. For clarity p-values (*** p<0.001) are indicated only for differences between *Stat1^ind^* cells pretreated with or without dox. Differences between *Stat1^−/−^* and untreated *Stat1^ind^* cells were significant (p<0.001) compared to WT and *Stat1^ind^* cells pretreated with dox for all mRNAs analyzed except for basal *Irf1* expression. **B**) PEFs were isolated from WT, *Stat1^−/−^* (*S1^−/−^*) and *Stat1^ind^* mice. 5×10^3^ cells were plated onto each well of a 96-well plate, pretreated with 0.025 µg/ml dox for 24 h, stimulated with serial 2-fold dilutions of IFNγ starting at 100 U/ml for 24 h and infected with VSV at a moi of 0.5. After two or three days surviving cells were stained with crystal violet (left panel). Stained cells were solubilized and crystal violet intensity was measured at 595 nm. Resulting values were calculated relative to each untreated genotype. Mean values ± SE from three independent experiments are shown (right panel). **C–E**) BMMΦs were isolated from WT and *Stat1^ind^* mice, pretreated with increasing amounts of dox (0.1, 0.125, 0.15, 0.25 and 10 µg/ml) for 24 h and stimulated with 100 U/ml IFNβ or IFNγ for 4 h or left untreated. RNA was isolated and cDNA used to analyze expression of *Stat1* (C), *Mx1* (D) and *Irf1* (E) mRNA as described in (A).

WT, *Stat1^−/−^* and *Stat1^ind^* BMMΦs were pre-treated with dox and stimulated with IFNβ or IFNγ. Dox-treated *Stat1^ind^* and WT cells have similar levels of *Stat1* mRNA (Fig. 3A, upper panel). The expression levels of *Mx1* in *Stat1^−/−^* cells and untreated *Stat1^ind^* cells were below detection limits but upon treatment with IFNβ *Mx1* was strongly and similarly upregulated in WT and dox-treated *Stat1^ind^* cells, whereas it was only weakly induced in *Stat1^−/−^* and dox-untreated *Stat1^ind^* cells. As expected, IFNγ-triggered expression of *Mx1* was lower than when cells were stimulated with IFNβ, but was comparable between WT- and STAT1^FLAG^-expressing cells (Fig. 3A, middle panel). Similar results were found when the expression of *Irf1* was investigated. Only dox-treated *Stat1^ind^* cells showed *Irf1* induction upon IFNβ or IFNγ stimulus, with the level of induction comparable to that seen in WT cells (Fig. 3A, lower panel). The data demonstrate the transcriptional activity of STAT1^FLAG^, which readily reaches WT levels upon IFN treatment.

STAT1 is essential in IFNγ-mediated defense against Vesicular Stomatitis Virus (VSV) [20]. PEFs from WT, *Stat1^−/−^* and *Stat1^ind^* mice were pretreated with dox, stimulated with serial 2-fold dilutions of IFNγ and either challenged with VSV or left untreated. Cells expressing STAT1^FLAG^ showed similar resistance against the virus to WT cells, while *Stat1^−/−^* cells were highly susceptible (Fig. 3B). Consistent with the basal level of STAT1^FLAG^ expression in *Stat1^ind^* PEFs in the absence of dox, these cells showed some antiviral activity, although less than WT. BMMΦs are highly resistant against VSV infections due to their higher levels of endogenous IFN type I [54]. WT and *Stat1^ind^* BMMΦs pretreated with dox survived VSV challenge, whereas *Stat1^ind^* BMMΦs without dox did not confer any antiviral activity (Fig. S3). STAT1^FLAG^ conferred resistance against VSV, as expected from the IFN-induced expression of ISGs.

### Minute amounts of STAT1 are sufficient to induce WT transcriptional responses in BMMΦs

The expression of STAT1 varies between cell types [55] but little is known about the quantity of STAT1 required for full transcriptional activation of ISGs. We analyzed IFN-dependent expression of *Mx1* and *Irf1* in cells provided with different amounts of STAT1^FLAG^. Different amounts of dox resulted in different levels of *Stat1^ind^* mRNA (Fig. 3C) and STAT1^FLAG^ protein (Fig. S4). *Stat1* levels considerably lower than those found in WT (Fig. 3C) are sufficient to induce WT levels of *Mx1* (Fig. 3D) and *Irf1* (Fig. 3E) after stimulation with IFNβ or IFNγ. Hence, only a fraction of the STAT1 provided in WT is able to reconstitute a full transcriptional response in BMMΦs.

### STAT1^FLAG^ is widely expressed *in vivo*

**Figure 4:**
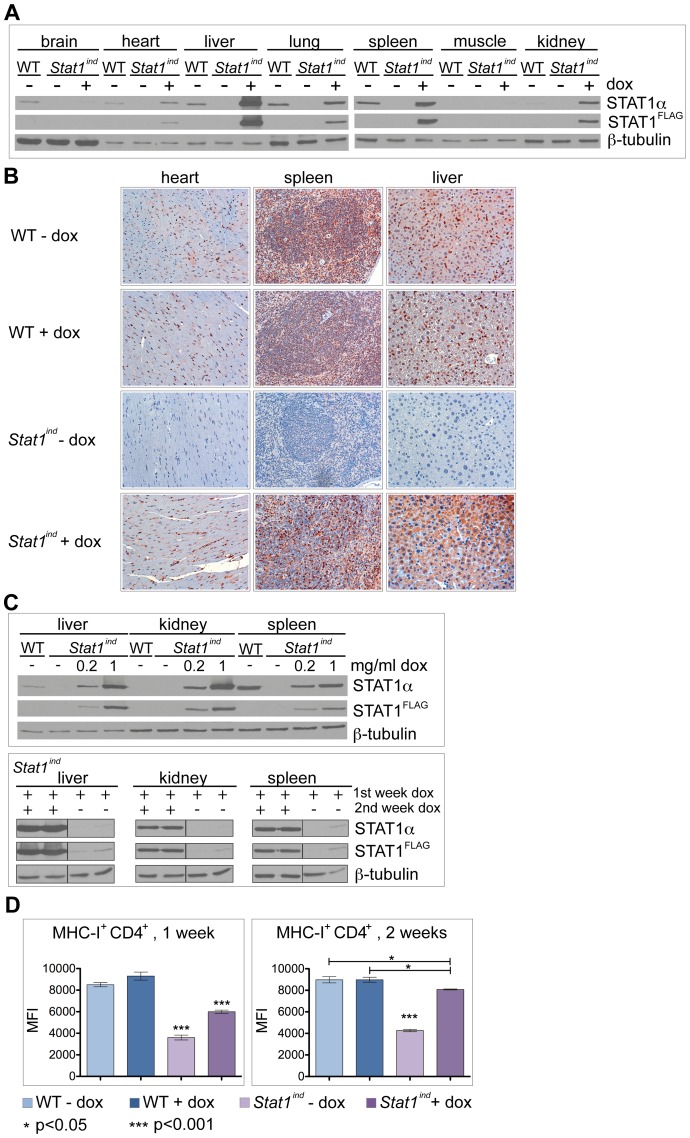
STAT1^FLAG^ levels in organs and MHC-I expression on splenocytes. **A, C**) WT and *Stat1^ind^* mice were treated with (i) 1 mg/ml dox in the drinking water for three days (A), (ii) 0.2 mg/ml or 1 mg/ml dox for two weeks (C, upper panel) or (iii) for 1 week with 1 mg/ml dox and in the second week with (+) or without (−) dox (C, lower panel). Brain, heart, liver, lung, spleen, muscle and kidney were isolated and protein extracts used for Western blot analysis. Antibodies against STAT1α and FLAG were used, with β-tubulin as loading control. **B**) WT and *Stat1^ind^* mice were treated with 1 mg/ml dox in the drinking water for two weeks or left untreated. Heart, spleen and liver were removed and immunohistochemistry performed to analyze STAT1 expression. One representative picture from one of three mice per group is shown. **D**) Mice of the indicated genotypes were treated for one or two weeks with 1 mg/ml dox *via* the drinking water. FACS was used to examine the surface expression levels of MHC-I (H-2Db) on freshly isolated splenocytes. The average level of H-2Db (MFI±SD) in CD4^+^ T cells is shown. n = 3 for all genotypes, except for *Stat1^ind^* without dox after one week treatment (n = 2). P-values between different groups are indicated; p-values above bars indicate significant differences to all other groups compared (* p<0.05; *** p<0.001).

To examine the *in vivo* expression of STAT1^FLAG^, WT and *Stat1^ind^* mice were treated with dox for 3 days and levels of STAT1 protein in various organs analyzed by Western blotting (Fig. 4A and C). In WT mice, the highest amounts of STAT1 were detected in the spleen, with liver and lung also showing high levels. WT STAT1 expression was lower in brain and heart and hardly detectable in kidney and muscle. STAT1^FLAG^ expression was lower compared to WT in brain, similar to that of WT in heart, lung, spleen and muscle, and liver and kidney showed higher levels of STAT1^FLAG^ than of WT STAT1. Analysis of *Stat1* mRNA expression revealed that transcription and translation grossly correlate with minor differences observed in heart and spleen (Fig. S5).

Immunohistochemistry was used to obtain more detailed information about the distribution of STAT1^FLAG^ and WT STAT1 in various organs. STAT1^FLAG^ was not detectable in heart, spleen and liver of *Stat1^ind^* mice in the absence of dox (Fig. 4B, third row). In skin of *Stat1^ind^* mice we detected some mesenchymal, fibroblast-like cells expressing STAT1^FLAG^ also in the absence of dox (Fig. S6, third column). This goes in line with the basal expression of STAT1^FLAG^ observed in PEFs (Fig. S2B). Dox treatment of WT mice had no effect on STAT1 expression (Fig. 4B, first two rows). In spleen and heart, the levels and distribution of STAT1 were similar in WT and dox-treated *Stat1^ind^* mice (Fig. 4B, first two columns). In the liver, WT STAT1 was found in Kupffer cells with only no or very low amounts in the hepatocytes, consistent with the notion that different cell types may have different basal levels of STAT1. In dox-treated *Stat1^ind^* mice, STAT1^FLAG^ was readily detectable in hepatocytes but not in Kupffer cells (Fig. 4B, right column). Different expression levels of STAT1^FLAG^ compared to WT STAT1 in brain, liver and kidney can be explained by the bioavailability of dox in different cells/organs and different dox-detoxification rates *in vivo*. Since *Stat1^ind^* expression is not driven by the endogenous *Stat1* promoter, the accessibility of the *Rosa26* and the *Col1a1* locus may also contribute to different *Stat1^ind^* expression levels in different cell types. However, the results are consistent with previous reports that the *Rosa26* locus drives widespread expression of the tet-activator [46].

### STAT1^FLAG^ expression *in vivo* is dose-dependent and reversible

To examine the dose dependence of STAT1 functions *in vivo*, we applied two different concentrations of dox (0.2 and 1 mg/ml) in the drinking water. The lower dose resulted in lower levels of STAT1^FLAG^ in liver, kidney and spleen (Fig. 4C, upper panel). Dox withdrawal resulted in a significant reduction of STAT1^FLAG^ after one week (Fig. 4C, lower panel). This proves that *Stat1^ind^* mice express STAT1^FLAG^ in a dose-dependent and reversible manner, so they will be useful for examining STAT1 functions that are time-restricted.

### STAT1^FLAG^ reconstitutes MHC-I homeostasis on CD4^+^ and CD8^+^ splenocytes

Previous studies have shown that homeostatic MHC-I on B and T cells is dependent on STAT1 [56]. To analyze MHC-I expression in splenocytes *in vivo*, we treated WT and *Stat1^ind^* mice with dox for one or two weeks. MHC-I expression was clearly reduced on CD4^+^ T cells in *Stat1^ind^* mice without dox (Fig. 4D) as previously shown for *Stat1^−/−^* mice [56]. Dox treatment for one or two weeks resulted in STAT1^FLAG^-mediated expression of MHC-I on CD4^+^ cells and after two weeks of treatment expression was almost at WT levels (Fig. 4D). Similar results were obtained in CD8^+^ T-cells (Fig. S7). STAT1^FLAG^ is thus able to restore homeostatic MHC-I levels *in vivo.*

### STAT1^FLAG^ confers antiviral activity against VSV and EMCV *in vivo*

**Figure 5:**
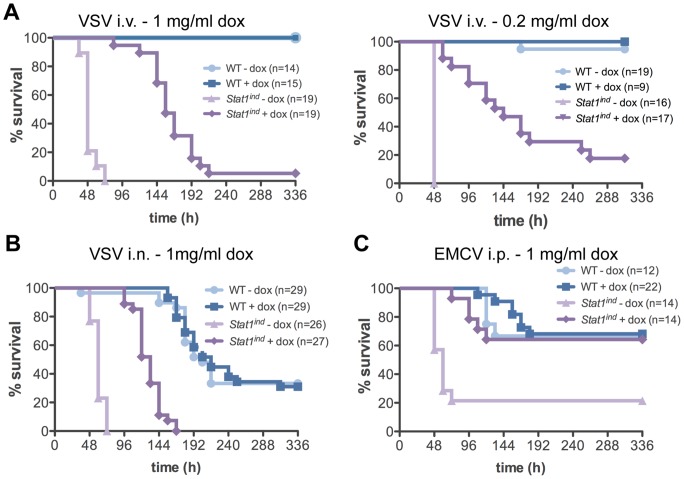
STAT1^FLAG^-mediated defense against VSV and EMCV infections. WT and *Stat1^ind^* mice were pretreated with the indicated concentrations of dox in the drinking water for one week or left untreated **A**) Mice were injected with 10^5^ pfu/mouse VSV intraveniously (i.v.) and survival monitored for two weeks. Numbers of mice are indicated and results are shown from four (left panel) or two (right panel) independent experiments. There was no significant difference in survival between WT mice treated with or without dox. The survival of *Stat1^ind^* mice with or without dox was significantly different (p<0.0002) from that of all other groups. **B**) Mice were injected with 10^5^ pfu/mouse VSV intranasally (i.n.) and survival monitored for two weeks. Results are shown from three independent experiments. The survival rate of *Stat1^ind^* mice without dox and *Stat1^ind^* mice with dox was significantly different (p<0.0001) from that of all other groups. **C**) Mice were injected with 5 pfu/mouse EMCV intraperitoneally (i.p.) and survival monitored for two weeks. Results are shown from two independent experiments. The survival of *Stat1^ind^* mice without dox was significantly different (p<0.0024) from that of all other groups.

*Stat1*-deficient mice are highly susceptible to VSV, which is mainly cleared through IFN type I activity [19], [20], [57]. Intravenous (i.v.) challenge with VSV results in high viral loads in spleen and liver [57]. Mice were pretreated with 1 mg/ml dox to give similar or higher STAT1 levels in liver and spleen to those found in WT animals and challenged one week later i.v. with VSV. All WT mice survived, whether or not they were treated with dox. All untreated *Stat1^ind^* mice succumbed to VSV infection within 72 h, as previously shown for *Stat1^−/−^* mice [19], [20] (Fig. 5A, left panel). STAT1^FLAG^ expression in mice conferred significantly higher survival upon VSV infection showing that STAT1^FLAG^ is functional *in vivo*. However, compared to WT mice the survival was lower. Lowering the dose of dox to 0.2 mg/ml resulted in decreased levels of STAT1^FLAG^ in spleen and liver. No significant differences in survival were observed between mice treated with high (Fig. 5A left panel) and low (Fig. 5A, right panel) amounts of dox., i.e. lower levels of STAT1 prior to infection do not impair STAT1-mediated antiviral activity and survival against VSV.

To assess the anti-VSV potency of STAT1^FLAG^ in another organ subject to primary infection, we inoculated mice intranasally (i.n.) with VSV. This route of infection leads to infection of the CNS and gives a lethality 3–4 orders of magnitude higher than found with systemic infection [58], [59]. VSV challenge *via* this route resulted in 40% survival of WT mice (compared to 100% after i.v. challenge, Fig. 5A and B). As after i.v. application of the virus, *Stat1^ind^* mice without dox succumbed to the infection within 48 h. Again, reconstitution with STAT1^FLAG^ led to improved resistance against VSV. It should be noted that resistance remained lower than in WT animals: the difference does not stem from toxicity of overexpressed hepatic STAT1^FLAG^, as WT and *Stat1^ind^* livers show no pathomorphological differences on day 5 p.i. (Fig. S8A) and high levels of STAT1^FLAG^ do not lead to higher expression of ISGs (Fig. S8B).

Finally we tested the resistance to Encephalomyocarditis Virus (EMCV) infection. Intraperitoneal (i.p.) infection with EMCV leads to acute myocarditis and encephalitis and high viral titers in brain, heart, liver and spleen [60], [61]. Mice deficient for *Ifnar1* or *Stat2* are highly susceptible to EMCV, indicating that a functional IFN type I response is essential for host defense [62], [63]. We infected dox-pretreated mice i.p. with EMCV (5 pfu/mouse) and monitored survival for 14 days. In the absence of dox, 80% of *Stat1^ind^* mice succumbed to the infection within 48 h, whereas in the presence of dox the survival rate was similar to that of WT mice at about 70% (Fig. 5C). STAT1^FLAG^ is thus able to mediate antiviral immunity.

## Discussion

Here we report the generation of *Stat1^ind^* mice based on a Tet-On [64], i.e. dox-inducible, STAT1 expression system, in which the transgene components are integrated into defined loci that confer sufficient, stable and ubiquitous expression (Fig. 1A and [46]). *Stat1^ind^* mice are homozygous triple mutants, with insertion of the tet activator at the *Rosa26* locus, insertion of the dox-dependent *FLAG*-tagged *Stat1* construct at the *Col1a1* locus and inactivation of the endogenous *Stat1* locus. In the uninduced state, *Stat1^ind^* mice are a phenocopy of *Stat1^−/−^* mice: they are viable and show no obvious abnormalities (Fig. 4 and 5, *Stat1^ind^* – dox; [19]). Primary BMMΦs and PEFs show dox dose-dependent inducibility of STAT1^FLAG^ to a level clearly exceeding that provided by WT cells (Fig. 1B and Fig. S2B). Biochemical and biological activity of STAT1^FLAG^ upon IFN stimulation was assessed by phosphorylation-dependent DNA binding, induction of ISGs and antiviral function and found to be similar to that of WT (Fig. 2 and 3). An interesting aspect of our *in vitro* studies is the observation that BMMΦs expressing very low amounts of STAT1^FLAG^ are able to induce ISGs similarly to WT cells upon IFN treatment (Fig. 3C–E). Reconstitution studies of *Stat1*-deficient cells are usually performed with transient or stable genetic complementation assays using constitutive expression constructs and are not designed to determine the minimal amount of STAT1 required for full biological activity. The *Stat1^ind^* cells provide a suitable tool to study the impact of IFN dose and cell type on ISG expression. In addition they will allow us to address functions of the extranuclear and unphosphorylated STAT1 (U-STAT1) pools: transcriptional complexes containing U-STAT1 are thought to contribute to the delayed phase of the IFN response [65], [66]. U-STAT1 functions can be studied *in vitro* and *in vivo* by applying dox in the absence of cytokine (e.g. IFN) and/or by increasing dox dose in the presence of minute amounts of cytokine. With regard to STAT1-isoform specific functions, it has been postulated that the transcriptional inactive STAT1β acts as a dominant negative factor on STAT1α in IFNγ signaling [67]. We did not observe excessive transcriptional or antiviral activity upon IFNγ treatment in cells expressing STAT1α only.

*In vivo* dox-induced STAT1^FLAG^ was detected in all organs analyzed (Fig. 4). We could not detect transgenic STAT1 in the absence of dox in cells (apart from fibroblasts), tissues or whole organs (apart from heart), confirming the tightness of the system [46], [47]. Dox-induced expression of STAT1^FLAG^ *in vivo* resulted in levels of STAT1 that were similar, higher or lower to those seen in the WT, depending on the tissue/organ analyzed and the dose of dox administered. STAT1^FLAG^ expression was not always comparable to that of WT-STAT1 in all organs – most likely a result of different accessibility of the *Col1a1* and the *Rosa26* locus and varying disposability of dox in different organs. Since COL1A1 is highly expressed in fibroblasts, we speculate that the observed leakiness of the system in this cell type is due to residual transcription of the *Stat1^ind^* construct from the *Col1a1* locus [55], [68]. While the recently described conditional *Stat1* mice [28], [50] allow the irreversible manipulation of STAT1 expression, *Stat1^ind^* mice facilitate time-restricted STAT1^FLAG^ expression by simple deprivation of dox. Nevertheless, it should be noted that, depending on the tissues or organs studied, dox deprivation requires periods of up to 4 weeks and even thereafter low-level release from depots has been reported [69], [70]. Tissue-specific production of STAT1^FLAG^ can be readily achieved by replacing the *Rosa26*-controlled tet transactivator by cross-breeding with one of the various tissue-/cell-type-specific rtTA mice [71].

Survival upon challenge with viruses revealed that levels of STAT1^FLAG^ suffice to confer WT response to EMCV, while upon VSV infection mice expressing dox-induced STAT1^FLAG^ performed significantly better than untreated controls, although their survival rates did not reach WT levels (Fig. 5). In experimental settings the two viruses have different primary target organs and pathogenic mechanisms as well as differing molecular interfaces with the host immune system [60], [72]–[74]. To clear the infection in each case the host depends on a functional IFN-I response, and hence on STAT1, although the temporal and spatial STAT1 activities required for host survival are unknown.

The reasons for the failure of STAT1^FLAG^ to clear VSV infection completely under our experimental conditions most likely relate to inappropriate levels of STAT1 in particular organs and tissues and/or to unbalanced STAT levels in a given cell type. As mentioned above, in view of the pharmacodynamics of dox after oral application [75], [76] and of the variations in the chromatin accessibility of the transgene-controlling loci between various tissues and stages, it is not to be expected that STAT1^FLAG^ levels at a given dose and route of application of dox will match the levels of WT STAT1 in all organs and tissues [77]. It should be noted that STAT1^FLAG^ is massively overexpressed in hepatocytes but, in contrast to WT, not detectable in Kupffer cells. Hepatic STAT1 expression is not toxic, as we did not see any obvious changes in immunohistochemistry (Fig. S8A). Tests of the hepatic expression of ISGs showed similar amounts of *Mx1* and *Irf1* mRNA in mice expressing WT STAT1 and STAT1^FLAG^ after 5 days of VSV infection. Notably, overexpressed STAT1^FLAG^ did not translate to higher levels of ISGs (Fig. S8B). In addition, reconstitution with STAT1^FLAG^ led after two weeks to restoration of WT MHC-I expression on splenocytes. It is conceivable that restoring the chromatin landscape of the MHC-I locus and probably also of other important loci for innate and adaptive immunity might be more complex and time-consuming than restoring STAT1 levels [16].

The importance of balanced cellular STAT levels has been shown for infection with Lymphocytic Choriomenengitis Virus (LCMV): the ratios of basal and induced STAT1/STAT4 determine the innate and adaptive immune cell activities [14], [15]. Imbalances in the cellular STAT ratio in dox-treated *Stat1^ind^* mice might therefore contribute to the incomplete defense against VSV. Future studies will address these issues and determine the dose, application route and duration of dox treatment required to generate appropriate levels of STAT1 in organs/cell types of interest.

In summary, we report the generation of the first functional, regulatable and reversible *Stat1* transgenic mouse that enables the study of STAT1 functions under conditions of under- and overexpression or of normal expression, in the phosphorylated or unphosphorylated state. The *Stat1^ind^* mice will serve as a powerful tool to study the contribution of STAT1 to the onset, progression and outcome of disease.

## Supporting Information

Figure S1 **Southern Blot analysis of ES cells targeted with a dox-inducible** ***Stat1*** **construct.** **A**) Schematic organization of the *Col1a1* locus in WT (upper panel), KH2 (middle panel) and *Stat1^ind^* cells (lower panel). Open boxes represent the last exons of the *Col1a1* gene. SpeI restriction sites, the probe used for Southern blot analysis and resulting fragments are indicated. pA: polyadenylation signal; *frt*: flippase recognition target; P: promoter; neo: neomycin resistance cassette; hygro: hygromycin resistance cassette; SA: splice acceptor; tetO: tetracycline operator. **B**) DNA was isolated from WT, KH2 and *Stat1^ind^* ES cells and digested with SpeI and Southern blotting was performed using a probe against the *Col1a1* locus. Fragments of 6.2 kb refer to WT DNA; KH2 cells harboring frt sites result in fragments of 6.7 kb (frt); and *Stat1^ind^* DNA gives rise to a 4.1 kb fragment (flp-in).
(TIF)

Figure S2 **Dox time- and dose-dependent expression of STAT1^FLAG^ in ES cells, PEFs and splenocytes.** **A**) *Stat1^ind^* ES cells were treated with 0.1, 1 or 10 µg/ml dox for 24 h or left untreated. PEFs (**B**) and splenocytes (**C**) were isolated from *Stat1^ind^* mice and dox-treated with different amounts for 24 h (upper panels) or for different times (lower panels). **A, B, C**) Western blot was performed to analyze the expression of STAT1, membranes were reprobed with a FLAG-specific antibody. Loading was controlled with panERK or β-tubulin. Splenocytes were isolated from whole spleens mashed through a 100 µm cell strainer and red blood cells removed using Red Blood Cell Lysis Buffer (Sigma). Splenocytes were grown for 5 days in RPMI medium supplemented with 10% FCS, 2 mM L-Glutamin, Penecillin/Streptomycin (100 μg/ml and 100 U/ml), 50 μM β-Mercaptoethanol and 2 μg/ml Concanavalin A (all Sigma).
(TIF)

Figure S3 **Antiviral activity of STAT1^FLAG^ in BMMΦs.** BMMΦs were isolated from WT and *Stat1^ind^* mice and 4×10^4^ cells plated onto each well of a 96-well plate. Cells were treated with 0.25 µg/ml dox for 48 h and subsequently infected with serial 2-fold dilutions of VSV starting at a moi of 100. After 40 h surviving cells were stained with crystal violet.
(TIF)

Figure S4 **Dox dose-dependent expression of STAT1^FLAG^ in BMMΦs.** BMMΦs were isolated from WT and *Stat1^ind^* mice and stimulated with indicated amounts of dox for 24 h or left untreated. Protein lysates were used to perform WB to analyze STAT1α and STAT1^FLAG^ expression, panERK was used as loading control. One representative blot from three independent experiments is shown.
(TIF)

Figure S5 ***Stat1^ind^*** **expression in organs.** WT and *Stat1^ind^* mice were treated with 1 mg/ml dox in the drinking water for three days. RNA was isolated from brain heart, liver, lung, spleen, muscle and kidney and cDNA used to analyze *Stat1* expression. *Ube2d2* was used for normalization and expression values were calculated relative to each untreated WT organ. Results are shown as mean values ± SE from three animals per genotype and treatment from two independent experiments. P-values above bars indicate significant differences compared to all other groups (* p<0.05; **p<0.01; *** p<0.001).
(TIF)

Figure S6 **STAT1^FLAG^ expression in skin.** WT and *Stat1^ind^* mice were treated with 1 mg/ml dox in the drinking water for three days or left untreated. Skin was removed and immunohistochemistry performed to analyze STAT1 expression. One representative picture from one of two mice per group is shown.
(TIF)

Figure S7 **MHC-I expression on CD8^+^ splenocytes.** Mice of the indicated genotypes were treated for one or two weeks with 1 mg/ml dox *via* the drinking water. FACS was used to examine the surface expression levels of MHC-I (H-2Db) on freshly isolated splenocytes. The average level of H-2Db (MFI±SD) in CD8^+^ T cells is shown. n = 3 for all genotypes, except for *Stat1^ind^* without dox after one week treatment (n = 2). P-values between different groups are reported; p-values above bars indicate significant differences to all other groups compared (* p<0.05; **p<0.01; *** p<0.001).
(TIF)

Figure S8 **Expression of STAT1 protein and** ***Stat1*** **,** ***Mx1*** **and** ***Irf1*** **mRNA in liver of VSV-infected mice.** WT and *Stat1^ind^* mice were treated with 1 mg/ml dox for one week and subsequently injected i.v. with VSV (10^5^ pfu/mouse; +VSV) or as a control with PBS (−VSV). **A**) Liver was isolated on day 5 p.i. to analyze STAT1 expression by immunohistochemistry. One representative picture from one out of three mice per group is shown. **B**) Total RNA was isolated from liver 5 days p.i. and cDNA was used to analyze expression of *Stat1* (upper panel), *Mx1* (middle panel) and *Irf1* (lower panel) mRNA. Values were normalized to *Ube2d2* and calculated relative to PBS-treated WT mice. Results are shown as mean values ± SE; p-values (*** p<0.001) are indicated. n = 2 for PBS treated mice, n = 3 for VSV-treated mice.
(TIF)
