# Supplementary figures and images for: Inducible, Dose-Adjustable and Time-Restricted Reconstitution of Stat1 Deficiency In Vivo
Source: PLoS One. 2014 Jan 29;9(1):e86608. doi: 10.1371/journal.pone.0086608 (PMC3906053; doi:10.1371/journal.pone.0086608)

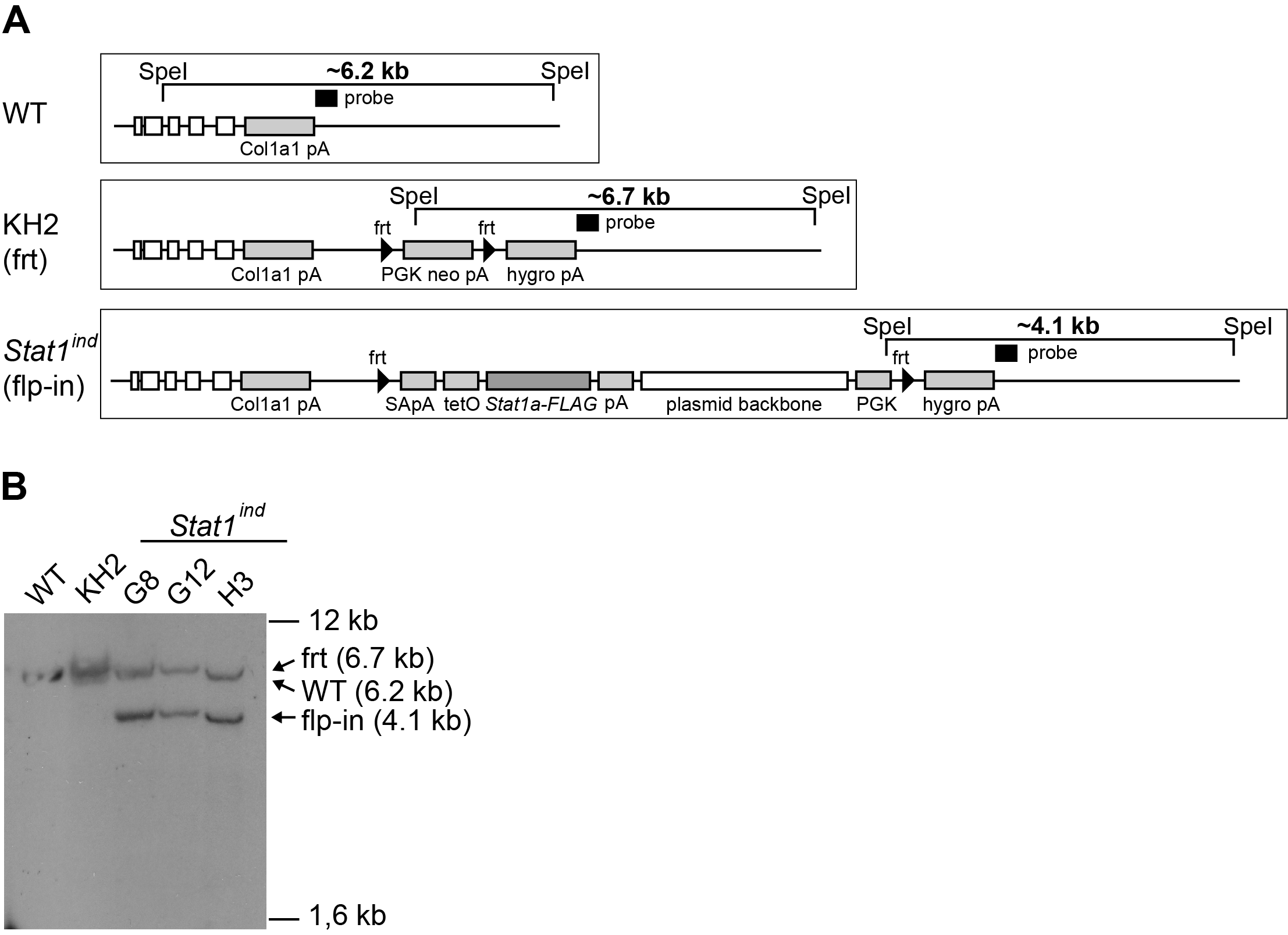

Supplement: Figure S1 — Southern Blot analysis of ES cells targeted with a dox-inducible Stat1 construct. A) Schematic organization of the Col1a1 locus in WT (upper panel), KH2 (middle panel) and Stat1ind cells (lower panel). Open boxes represent the last exons of the Col1a1 gene. SpeI restriction sites, the probe used for Southern blot analysis and resulting fragments are indicated. pA: polyadenylation signal; frt: flippase recognition target; P: promoter; neo: neomycin resistance cassette; hygro: hygromycin resistance cassette; SA: splice acceptor; tetO: tetracycline operator. B) DNA was isolated from WT, KH2 and Stat1ind ES cells and digested with SpeI and Southern blotting was performed using a probe against the Col1a1 locus. Fragments of 6.2 kb refer to WT DNA; KH2 cells harboring frt sites result in fragments of 6.7 kb (frt); and Stat1ind DNA gives rise to a 4.1 kb fragment (flp-in). (TIF) [file pone.0086608.s001.tif]

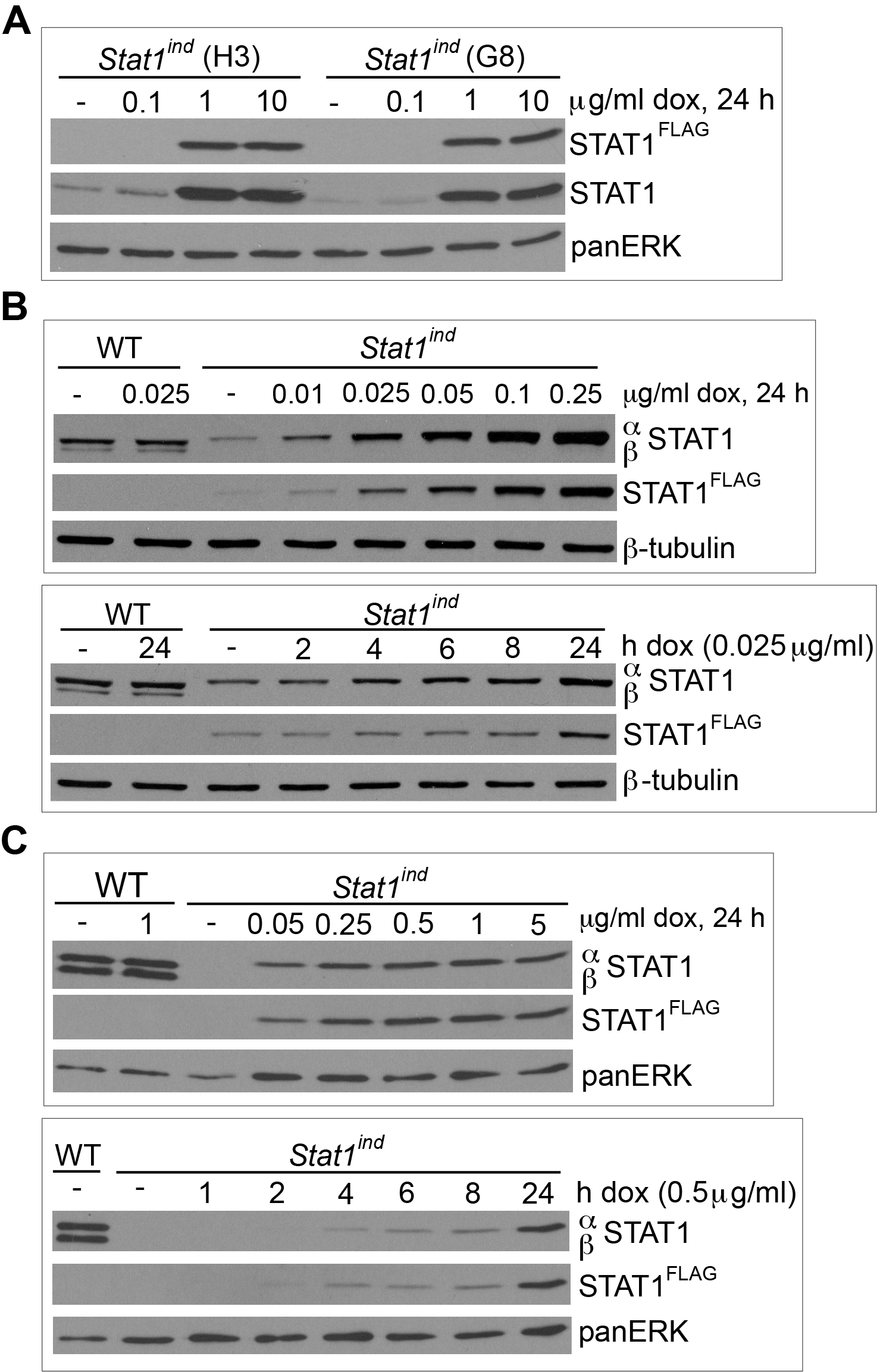

Supplement: Figure S2 — Dox time- and dose-dependent expression of STAT1FLAG in ES cells, PEFs and splenocytes. A) Stat1ind ES cells were treated with 0.1, 1 or 10 µg/ml dox for 24 h or left untreated. PEFs (B) and splenocytes (C) were isolated from Stat1ind mice and dox-treated with different amounts for 24 h (upper panels) or for different times (lower panels). A, B, C) Western blot was performed to analyze the expression of STAT1, membranes were reprobed with a FLAG-specific antibody. Loading was controlled with panERK or β-tubulin. Splenocytes were isolated from whole spleens mashed through a 100 µm cell strainer and red blood cells removed using Red Blood Cell Lysis Buffer (Sigma). Splenocytes were grown for 5 days in RPMI medium supplemented with 10% FCS, 2 mM L-Glutamin, Penecillin/Streptomycin (100 μg/ml and 100 U/ml), 50 μM β-Mercaptoethanol and 2 μg/ml Concanavalin A (all Sigma). (TIF) [file pone.0086608.s002.tif]

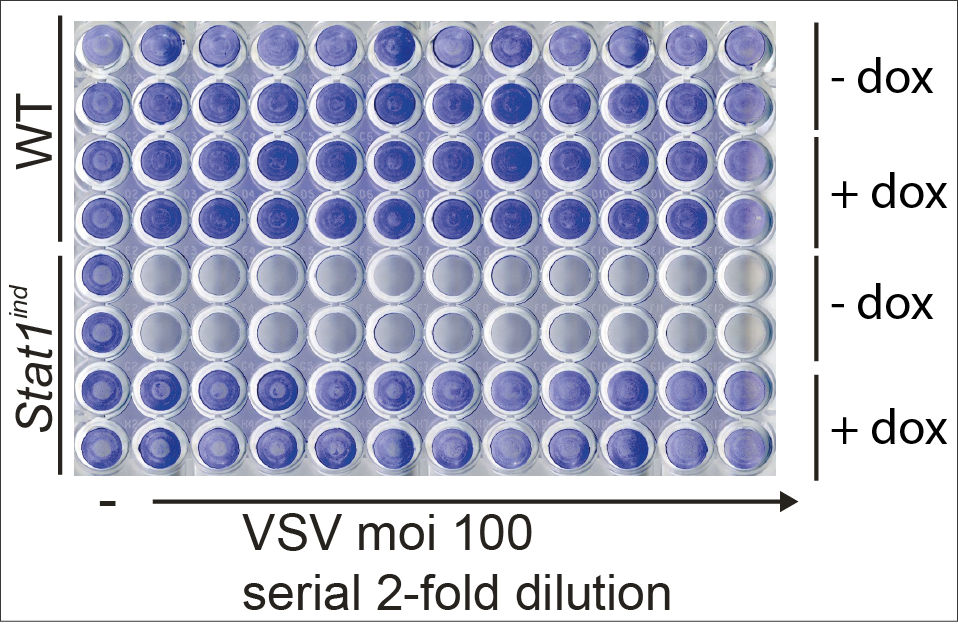

Supplement: Figure S3 — Antiviral activity of STAT1FLAG in BMMΦs. BMMΦs were isolated from WT and Stat1ind mice and 4×10^4^ cells plated onto each well of a 96-well plate. Cells were treated with 0.25 µg/ml dox for 48 h and subsequently infected with serial 2-fold dilutions of VSV starting at a moi of 100. After 40 h surviving cells were stained with crystal violet. (TIF) [file pone.0086608.s003.tif]

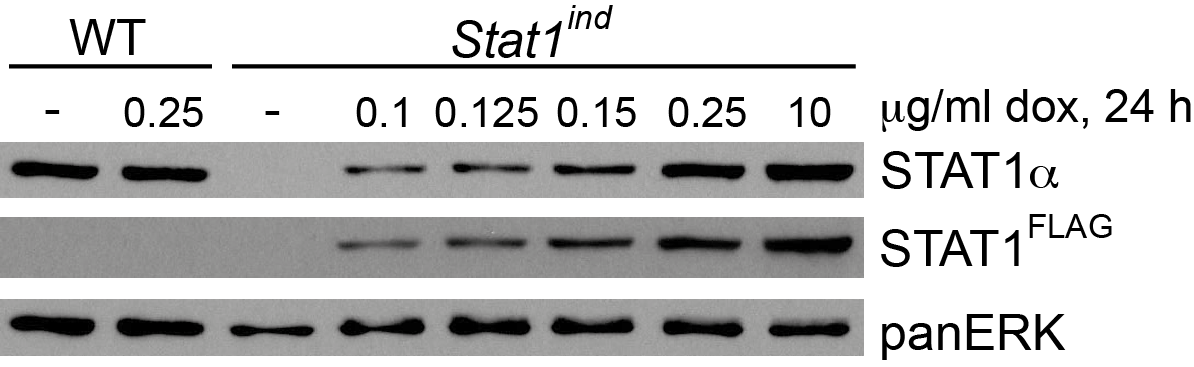

Supplement: Figure S4 — Dox dose-dependent expression of STAT1FLAG in BMMΦs. BMMΦs were isolated from WT and Stat1ind mice and stimulated with indicated amounts of dox for 24 h or left untreated. Protein lysates were used to perform WB to analyze STAT1α and STAT1FLAG expression, panERK was used as loading control. One representative blot from three independent experiments is shown. (TIF) [file pone.0086608.s004.tif]

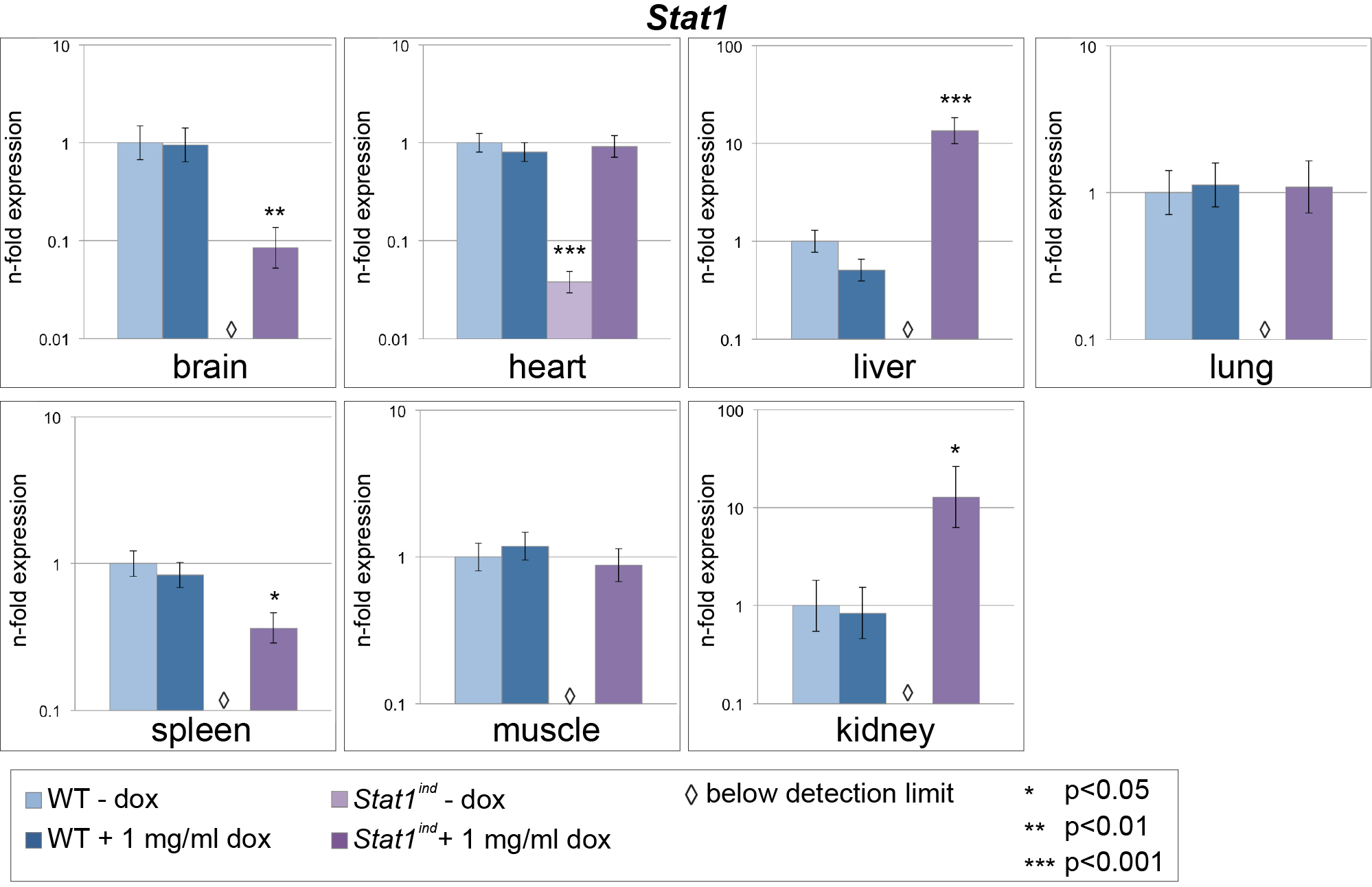

Supplement: Figure S5 — Stat1ind expression in organs. WT and Stat1ind mice were treated with 1 mg/ml dox in the drinking water for three days. RNA was isolated from brain heart, liver, lung, spleen, muscle and kidney and cDNA used to analyze Stat1 expression. Ube2d2 was used for normalization and expression values were calculated relative to each untreated WT organ. Results are shown as mean values ± SE from three animals per genotype and treatment from two independent experiments. P-values above bars indicate significant differences compared to all other groups (* p<0.05; **p<0.01; *** p<0.001). (TIF) [file pone.0086608.s005.tif]

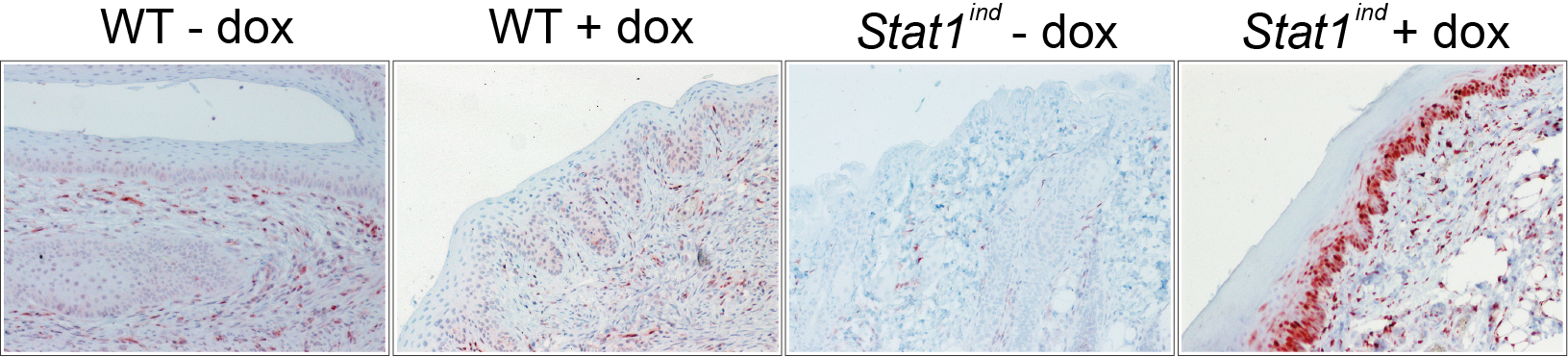

Supplement: Figure S6 — STAT1FLAG expression in skin. WT and Stat1ind mice were treated with 1 mg/ml dox in the drinking water for three days or left untreated. Skin was removed and immunohistochemistry performed to analyze STAT1 expression. One representative picture from one of two mice per group is shown. (TIF) [file pone.0086608.s006.tif]

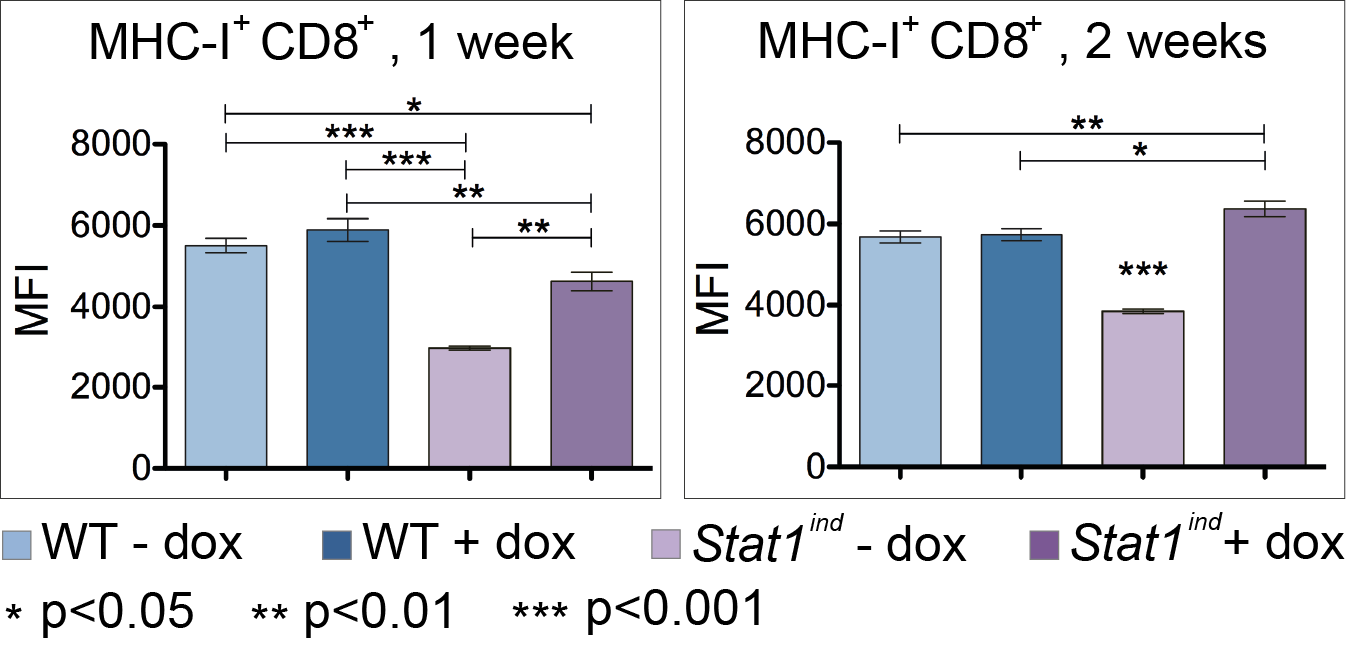

Supplement: Figure S7 — MHC-I expression on CD8+ splenocytes. Mice of the indicated genotypes were treated for one or two weeks with 1 mg/ml dox via the drinking water. FACS was used to examine the surface expression levels of MHC-I (H-2Db) on freshly isolated splenocytes. The average level of H-2Db (MFI±SD) in CD8+ T cells is shown. n = 3 for all genotypes, except for Stat1ind without dox after one week treatment (n = 2). P-values between different groups are reported; p-values above bars indicate significant differences to all other groups compared (* p<0.05; **p<0.01; *** p<0.001). (TIF) [file pone.0086608.s007.tif]

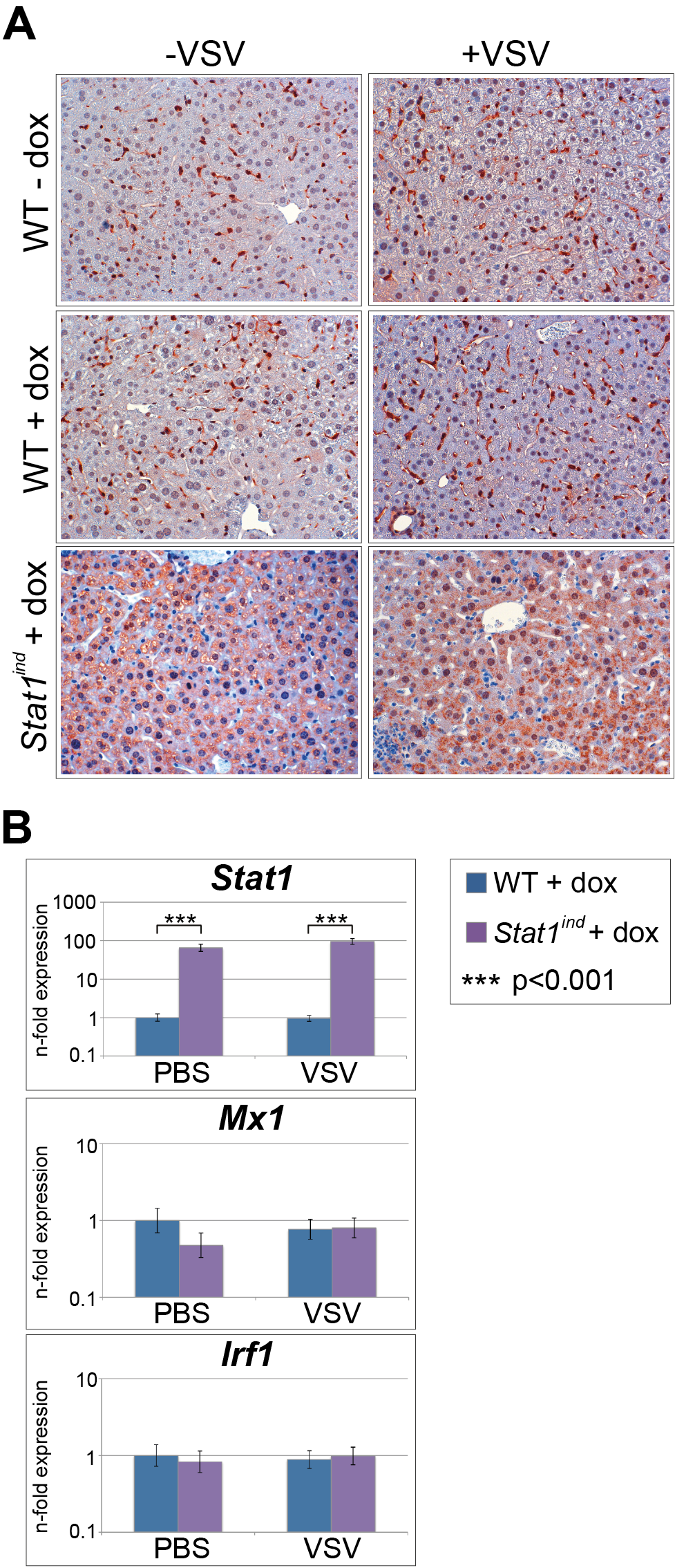

Supplement: Figure S8 — Expression of STAT1 protein and Stat1 , Mx1 and Irf1 mRNA in liver of VSV-infected mice. WT and Stat1ind mice were treated with 1 mg/ml dox for one week and subsequently injected i.v. with VSV (105 pfu/mouse; +VSV) or as a control with PBS (−VSV). A) Liver was isolated on day 5 p.i. to analyze STAT1 expression by immunohistochemistry. One representative picture from one out of three mice per group is shown. B) Total RNA was isolated from liver 5 days p.i. and cDNA was used to analyze expression of Stat1 (upper panel), Mx1 (middle panel) and Irf1 (lower panel) mRNA. Values were normalized to Ube2d2 and calculated relative to PBS-treated WT mice. Results are shown as mean values ± SE; p-values (*** p<0.001) are indicated. n = 2 for PBS treated mice, n = 3 for VSV-treated mice. (TIF) [file pone.0086608.s008.tif]
